# Wearable Ankle Robots in Post-stroke Rehabilitation of Gait: A Systematic Review

**DOI:** 10.3389/fnbot.2019.00063

**Published:** 2019-08-13

**Authors:** Bin Shi, Xiaofeng Chen, Zan Yue, Shuai Yin, Qipeng Weng, Xue Zhang, Jing Wang, Weina Wen

**Affiliations:** ^1^School of Mechanical Engineering, Institute of Robotics and Intelligent System, Xi'an Jiaotong University, Xi'an, China; ^2^Shaanxi Key Laboratory of Intelligent Robots, Xi'an, China; ^3^Baoxing Hospital, Shenzhen, China

**Keywords:** wearable ankle robots, actuator, gait event detection, control strategies, performance evaluation

## Abstract

**Background:** Stroke causes weak functional mobility in survivors and affects the ability to perform activities of daily living. Wearable ankle robots are a potential intervention for gait rehabilitation post-stroke.

**Objective:** The aim of this study is to provide a systematic review of wearable ankle robots, focusing on the overview, classification and comparison of actuators, gait event detection, control strategies, and performance evaluation.

**Method:** Only English-language studies published from December 1995 to July 2018 were searched in the following databases: PubMed, EMBASE, Web of Science, Scopus, IEEE Xplore, Science Direct, SAGE journals.

**Result:** A total of 48 articles were selected and 97 stroke survivors participated in these trials. Findings showed that few comparative trials were conducted among different actuators or control strategies. Moreover, mixed sensing technology which combines kinematic with kinetic information was effective in detecting motion intention of stroke survivors. Furthermore, all the selected clinical studies showed an improvement in the peak dorsiflexion degree of the swing phase, propulsion on the paretic side during push-off, and further enhanced walking speed after a period of robot-assisted ankle rehabilitation training.

**Conclusions:** Preliminary findings suggest that wearable ankle robots have certain clinical benefits for the treatment of hemiplegic gait post-stroke. In the near future, a multicenter randomized controlled clinical trial is extremely necessary to enhance the clinical effectiveness of wearable ankle robots.

## Introduction

Stroke is a leading cause of physical disability worldwide (Alguren et al., 2010). The absolute number of global stroke survivors reached 33 million in 2010, which has significantly increased by 84% since 1990 (Feigin et al., 2014). In a comprehensive prospective study of more than 800 stroke survivors, with a mortality rate of 21, 18% of stroke survivors were completely unable to walk, 11% of stroke survivors were able to walk with assistance, and 50% of stroke survivors were able to walk independently after rehabilitation (Jørgensen et al., 1995). The human ankle joint plays a key role in maintaining body balance while walking (Tejima, 2001). Impaired motor coordination (Cruz et al., 2009), muscle weakness and spasticity (Moriello et al., 2011), and reduced ankle dorsiflexion during walking are typical characteristics of post stroke gait, which restricts walking speed and causes gait compensations by hip hiking or circumduction of the paretic limb, increasing the risk of falling, and metabolic costs (Kerrigan et al., 2000; Chen et al., 2005; Cruz and Dhaher, 2009; Schmid et al., 2013; Susko et al., 2016). Gait abnormity of stroke survivors usually presents in various ways. Specifically, the reduction of plantar flexor and dorsiflexor are two typical characteristics of the ankle joints after stroke. On the one hand, weakness in the dorsiflexor muscles could manifest in an audible foot slap during the heel strike in the stance phase, and foot-drop and toe drag during the swing. On the other hand, weak plantar flexor muscles mainly affect lower-limb stability, and propulsion (Morris et al., 2011).

Conventional physical therapy mainly depends on the experience of the therapist, and it is very difficult to meet the requirements of high intensity, and repetitive training (Zhou et al., 2013). Ankle foot orthoses (AFO) are orthotic plastic devices which are externally applied to the ankle-foot joint to prevent foot-drop during the swing of walking (Alam et al., 2014). However, it inhibits normal push-off during walking (Vistamehr et al., 2014), and reduces gait adaptability (Van Swigchem et al., 2014). To facilitate ankle locomotion automatically and dynamically, in recent years, ankle rehabilitation robots have been developed to enable stroke survivors to regain walking capabilities. Ankle rehabilitation robots have proven to be an efficient technology in gait rehabilitation for stroke survivors (Zhang et al., 2013). Recent development in robot-assisted AFO demonstrates power assistance at the ankle joint and can facilitate walking of patients presenting with foot-drop, by actively assisting ankle dorsiflexion for foot clearance in the swing phase, and can minimize the occurrence of foot slap at initial contact (Dollar and Herr, 2008; Shorter et al., 2013; Alam et al., 2014). The high-intensity and repetitive nature of the robot promotes experience-driven adaptation of the damaged motor pathway in the CNS to the programmed gait pattern via brain plasticity (Landers, 2004; Moreno et al., 2013). A single-arm pilot study reported that stroke survivors (*n* = 8) had improved volitional ankle control and spatial-temporal gait parameters after 6 weeks with 18 sessions training using the Anklebot (Forrester et al., 2011). In summary, researches on experience-driven neuroplasticity suggest that to some extent, stroke survivors with foot-drop problems can potentially restore their walking ability through robot-assisted gait training with ankle dorsiflexion assistance on over-ground walking (Tucker et al., 2015).

Existing ankle rehabilitation robots could be categorized into platform-based ankle rehabilitation robots (Zhou et al., 2016; Liu et al., 2017) and wearable ankle rehabilitation robots. Wearable ankle rehabilitation robots can be defined as a wearable robotic device which actuates movement of the ankle joint and which can be used for over-ground walking with a programmable control. On the one hand, platform-based ankle rehabilitation robots are stationary robots whose goal is to move stroke survivors' ankle-foot to strengthen muscles, and to achieve motion therapy. On the other hand, wearable ankle rehabilitation robots are applied to the lower limb to offer plantar flexion/dorsiflexion, adduction/abduction, and inversion/eversion to perform gait training. In recent years, platform-based ankle robots were reviewed in studies (Miao et al., 2018; Zeng et al., 2018), as well as both platform-based ankle rehabilitation robots, and wearable ankle rehabilitation robots (Zhang et al., 2013; Jamwal et al., 2015; Khalid et al., 2015). However, they only discussed different designs, and control aspects of a few wearable ankle robots. To our knowledge, to date, a systematic overview of wearable ankle rehabilitation robots is lacking. Furthermore, the clinical effects of ankle rehabilitation robots for stroke survivors are not reviewed. Actuator, gait event detection, and control strategies are key factors for ankle rehabilitation robots, and have a great influence on the effectiveness of gait rehabilitation for stroke survivors. The actuator plays a curial role in the ankle rehabilitation robot and determines the assistive torque provided by the robot in gait training. Gait event detection, another core factor for gait rehabilitation of stroke survivors, can be used to trigger functional assistance. Control strategies are an integral part of the wearable ankle robot and aim to create a safe, comfortable, and natural human-computer interaction environment. Performance evaluations are often carried out with stroke survivors to quantitatively assess the therapeutic efficacy of the wearable ankle rehabilitation robot during gait training.

Therefore, in this paper, the current development of wearable ankle rehabilitation robots is systematically reviewed, focusing on the overview, classification, and comparison of actuator, gait event detection, control strategies and performance evaluation. This paper is organized into four sections with this section as the introduction. The methods used for literature is presented in section Methods. The results are described in detail in section Results where we review the actuator, gait event detection, control strategies, and clinical performance of wearable ankle rehabilitation robots. In section Discussion, we discuss the potential challenges of the above four topics and then provide our conclusions.

## Methods

Two authors (ZY and SY) conducted a literature search on 20 July 2018. Only English-language studies published from December 1995 to July 2018 were searched in PubMed, EMBASE, Web of Science, Scopus, IEEE Xplore, Science Direct, and SAGE journals. The keywords used for searching were “ankle” AND “rehabilitation” AND “robot.” Valuable references listed in relevant publications were further screened. This study aims to provide a systematic investigation of existing wearable ankle rehabilitation robots. Inclusion criteria consisted of studies involving wearable ankle rehabilitation robots. Exclusion criteria consisted of: (1) studies verifying the feasibility of ankle robots by simulation software; (2) studies involving passive ankle-foot orthoses; (3) studies involving hip or knee rehabilitation rather than ankle recovery; (4) studies that reduce the metabolic cost of human walking; (5) studies that strengthen the ankle joint by seated anklebot training instead of gait rehabilitation. The titles, abstracts and then the full text of the papers identified by the search were screened by two independent investigators (ZY and SY) according to the inclusion and exclusion criteria mentioned above. In the event of a conflict, a third reviewer (JW) was consulted for a resolution. Included articles were then examined to extract data regarding study design, exoskeleton devices, participant characteristics, intervention, training period, outcome measures, adverse effects, and results.

## Results

Initially 1,503 studies were identified. Forty-eight publications were included in the final review. A total of 19 studies assessed the effect of various ankle rehabilitation robots on impaired subjects, which included 97 stroke survivors, two plantar flexor impaired subjects, and four dorsiflexor impaired subjects. The study design included two RCT (Randomized Controlled Trial) designs, 15 case study designs and two case-control study designs.

**Figure 1:**
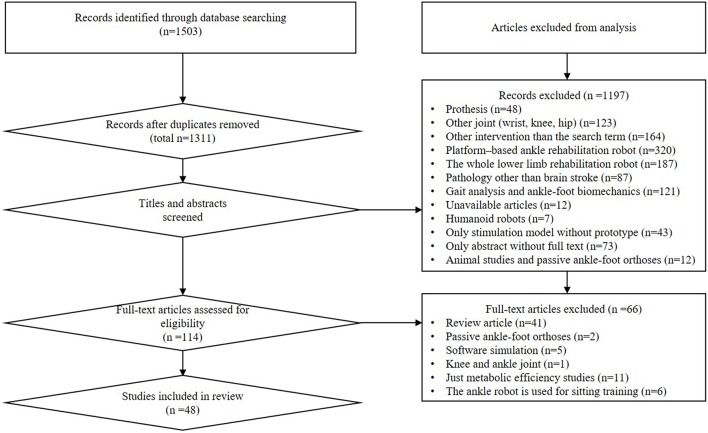
PRISMA flow chart of literature search procedures.

The schematic overview of selection process with the search results is shown in Figure 1.

### Classification of Wearable Ankle Robots

Wearable ankle rehabilitation robots known as a powered ankle exoskeleton or a powered ankle-foot orthoses are being developed (Hussain et al., 2017). In accordance with the FDA's definition, a robotic exoskeleton is a prescription device which consists of external and powered orthosis for medicine, and which is attached to a person's paralyzed or weakened limbs to assist with ambulation' (Food and Drug Administration, HHS 2015) (Contreras-Vidal et al., 2016). In this paper, based on structures, wearable ankle rehabilitation robots are mainly classified into soft powered ankle exoskeletons and rigid powered ankle exoskeletons.

#### Soft Powered Ankle Exoskeletons

A soft robotic exosuit designed by Harvard University was placed over the paretic limb to enhance forward propulsion and ground clearance, contributing to more normal walking post-stroke (Awad et al., 2017b). A bio-inspired soft wearable robotic device has been proposed by Carnegie Mellon University for ankle-foot rehabilitation (Park et al., 2014).

#### Rigid Powered Ankle Exoskeletons

An active AFO has been developed by the Massachusetts Institute of Technology (MIT) to assist foot-drop gait (Blaya and Herr, 2004). The powered exoskeleton has been proposed at North Carolina State University (Takahashi et al., 2015). An ankle rehabilitation robot was designed by the Chinese University of Hong Kong for robot-assisted gait training of stroke survivors (Yeung et al., 2018).

An overview of recent wearable ankle rehabilitation robots is listed in Table 1. Comparative analysis of the actuator, gait event detection, control strategies, and performance evaluation of the wearable ankle robots will be detailed in the next section in terms of their merits and demerits.

**Table 1:** Overview of recent wearable rehabilitation ankle robots.

**References**	**DoF**	**Weight**	**ROM**	**Actuator**	**Control strategies**	**Peak torque**	**Training modes**
Andersen and Sinkjaer, 1995	1	0.9 kg	Df/20° Pf	Motor actuator and bowden cable	Position control	218 Nm Pf/Df	Passive mode Assist mode
Blaya and Herr, 2004	1	2.6 kg	Df/27° Pf	SEA	Impedance control	/	Assist mode
Wheeler et al., 2004	3	3.6 kg	25° Df/45° Pf 25° Is/15° Es 15° Ad/15° Ab	Motor actuator	Position control	23Nm Pf/Df 15Nm Is/Es	Assist mode
Ferris et al., 2005	1	1.6 kg	Df/Pf	PMA	Proportional myoelectric control	38 Nm Df 70 Nm Pf	Assist mode
Cain et al., 2007	1	1.4 kg	Pf	PMA	Proportional myoelectric control Footswitch control	/	Assist mode
Ward et al., 2007	2	/	22.8° Df/22.9° Pf 3° Is/ 5° Es	SOM actuators	Position control	/	Assist mode
Kim et al., 2007	1	2.8 kg	11.9° Df/21.5° Pf	SEA	Phase-Based control	97.2 Nm Pf/Df	Assist mode
Ward et al., 2010	1	/	Df/Pf	SEA	Position control	20 Nm Pf/Df	Assist mode
Roy et al., 2013; Forrester et al., 2016	3	3.6 kg	25° Df/45° Pf 25° Is/20° Es 15° Ab/15° Ad	Motor actuator	Impedance control	23 Nm Pf/Df 15 Nm Is/Es	Assist mode
Tanida et al., 2009	1	1.1 kg	Df/Pf	MRFB	Force control	12 Nm Pf/Df	Resistive mode
Blanchette et al., 2014	1	1.7 kg	Df/Pf	Electro-hydraulic actuator	Force control	/	Assist mode
Shorter et al., 2011a	1	1.9 kg	30°Df/ Pf	Pneumatic rotary actuator	Position control	9 Nm Pf/Df	Assist mode
Park et al., 2014	2	0.95 kg	14° Df/13°Pf Is/Es	PMA	Position control	/	Assist mode
Takahashi et al., 2015	1	0.53 kg	Df/Pf	PMA	EMG control	/	Assist mode
Yeung et al., 2018	1	1.0 kg	20° Df/30° Pf	Motor actuator	Phase-based control	16.7 Nm Pf/Df	Assist mode
Awad et al., 2017b	1	0.9 kg	Df/Pf	Motor actuator	Position control	/	Assist mode
Choi et al., 2018	1	0.869 kg	Df/Pf	Motor actuator	Force control	20 Nm Pf/Df	Assist mode
Koller et al., 2018	1	2.08 kg	Pf	PMA	EMG control Finite state control	/	Assist mode

*DOF, degrees of freedom; SEA, series elastic actuator; PMA, pneumatic muscle actuator; MRFB, magnetorheologic fluid brake; EMG, electromyography; SOM, Spring over muscle; Df, Dorsiflexion; Pf, Plantar flexion; Is, Inversion; Es, Eversion; Ad, Adduction; Ab, Abduction*.

### Actuator

Common actuator modes of ankle robots are pneumatic muscle actuator (PMA), series elastic actuator (SEA), motor actuator, and hydraulic actuator. Based on the direction of the actuation, a power ankle exoskeleton could be divided into three distinctive groups as follows:

- Plantar flexion assistance devices;
- Dorsiflexion assistance devices;
- Plantar flexion/dorsiflexion assistance devices;

Plantar flexion assistance devices aim to reduce the user's metabolic cost, improve plantar flexion in push-off and further enhance walking speed. Furthermore, dorsiflexion assistance devices aim to prevent the forefoot from colliding with the floor at a high velocity in heel strike (i.e., foot slap), and the toes from hitting the floor during the swing (i.e., foot-drop). Plantar flexion/dorsiflexion assistance devices combine the functions of the above two devices.

#### PMA

The PMA consists of an inner layer made from butyl rubber tubing with two end-caps forming the terminal connectors to seal the muscle cylinder (Klute and Hannaford, 2000; Klute et al., 2002; Davis et al., 2003). The main merits of PMA are its high torque-to-weight ratio and natural compliance. PMA was applied to these studies (Bharadwaj et al., 2005; Ferris et al., 2006; Gordon et al., 2006; Sawicki et al., 2006; Cain et al., 2007; Ward et al., 2007; Kao et al., 2010; Park et al., 2014; Takahashi et al., 2015; Jacobs et al., 2018). Powered ankle exoskeleton based PMA are listed in Table 2.

**Table 2:** Overview of wearable ankle robot based PMA.

**References**	**Weight**	**Actuator**	**An/Posterior**	**Single/double**	**Control**	**DOF**	**Peak torque**
Ferris et al., 2005	1.6 kg	PMA	An/Posterior	Single	Tibialis anterior EMG Soleus EMG	Df/ Pf	38 Nm Df 70 Nm Pf
Ferris et al., 2006	1.7 kg	PMA	An/Posterior	Single	Tibialis anterior EMG Soleus EMG	Df/ Pf	20.7 Nm Df 50.7 Nm Pf
Sawicki et al., 2005	/	PMA	Posterior	Single	Soleus EMG	Pf	27 Nm Pf
					Footswitch	Pf	1.16 Nm/kg Pf
					Pushbutton (PC/TC)	Pf	/
Sawicki et al., 2006	1.1 kg	PMA	Posterior	Single	Pushbutton (PC/TC)	Pf	0.33 ± 0.02 Nm/kg Pf
Koller et al., 2015	2.08 kg	PMA	Posterior	Single	Soleus EMG	Pf	/
Koller et al., 2018					Soleus EMG Footswitch	Pf	/
Kao et al., 2010	1.1 kg	PMA	Posterior	Double	Soleus EMG	Pf	50.09 ± 12.05 Nm Pf
Kao and Ferris, 2009	/	PMA	Anterior	Single	Tibialis anterior EMG	Df	0.12 ± 0.09 Nm/kg Df
Bharadwaj et al., 2005; Ward et al., 2007	/	SOM	Anterior	Double	Position control	Df/ Pf Is/Es	/

*TC, therapist control; PC, patient control; Df, Dorsiflexion; Pf, Plantar flexion; Is, inversion; Es, eversion*.

A large proportion of developed ankle robots include unidirectional devices that provide only plantar flexion assistance (Sawicki et al., 2005, 2006; Gordon et al., 2006; Gordon and Ferris, 2007; Kao et al., 2010; Koller et al., 2015). In Kao et al. (2010), two PMAs were connected to the posterior of the orthosis to provide plantar flexion assistance. The maximum torque created by the ankle exoskeleton was 50.09 ± 12.05 Nm. Moreover, PMA was attached to the anterior of the orthosis to provide dorsiflexion assistance (Bharadwaj et al., 2005; Bharadwaj and Sugar, 2006; Ward et al., 2007). Specifically, a robotic gait trainer was proposed by Arizona State University to assist stroke survivors during walking (Ward et al., 2007). The RGT employs a pneumatic spring over muscle (SOM) to generate bidirectional forcing, which improves the shortage of PMA acted only in contraction forcing. The SOM actuator consists of a standard compression spring linked in parallel with a traditional pneumatic muscle and has bi-directional, compliant, and lightweight characteristics. When the two SOM actuators move in the simultaneous direction, the ankle is driven in plantar flexion/dorsiflexion in the sagittal plane. Similarly, the ankle is moved by the two SOM actuators in the opposing motion to generate inversion/eversion in the frontal plane. Two pneumatic muscles actuators were attached to the anterior and posterior shank sections to assist with plantar flexion and dorsiflexion (Ferris et al., 2005, 2006).

In Park et al. (2014), a bio-inspired soft wearable robotic device powered by four PMAs was used to assist dorsiflexion, plantar flexion, inversion, and eversion. The design mimics the morphology and functionality of the biological muscle-tendon-ligament structure to provide active assistance. This bio-inspired design is lightweight, providing multi-DOFs assistance, and natural degrees of freedom are not limited. However, the disadvantage of the device is that it is complex and not portable. Moreover, the details regarding the assistance torque were not reported in the study.

#### SEA

The series elastic actuator (SEA) is composed of a traditional brushless direct current (DC) motor in series with a standard spring (Veneman et al., 2016). To control the impedance of the orthotic ankle joint for sagittal plane rotations, Robinson et al. (1999) previously developed the SEA, which is used for lower-limb robots. Advantages of the SEA include: (1) it has low impedance; (2) it has high control precision and storing energy; (3) the effects of backlash, torque ripple, and friction are filtered by the spring; (4) the shock load is isolated from the motor (Pratt and Williamson, 1995). SEA is limited by its large volume, heavy mass, and complicated structure (Zhang et al., 2017).

An active ankle-foot orthosis was designed by the Massachusetts Institute of Technology (MIT) to provide motions in plantar flexion/dorsiflexion and to mainly prevent foot-drop and foot slap during walking (Blaya and Herr, 2004). Its total weight is 2.6 kg. It does however, have some disadvantages such as being tethered and being heavy. An active ankle-foot orthosis (AAFO) has also been developed at the Yonsei University (maximum torque 97.2 Nm plantar flexion/dorsiflexion) to help avoid foot slap during weight acceptance and foot-drop in the swing phase (Hwang et al., 2006; Kim et al., 2007, 2011). The active ankle-foot orthosis was powered by the SEA to provide for plantar flexion/dorsiflexion. The MIT's AFO and AAFO designed by Yonsei University have similar designs, with a SEA actuator attached posteriorly to a conventional AFO. A portable robotic device has been developed by Arizona State University to improve gait kinematics and to enhance gait speed and walking duration for stroke survivors (Ward et al., 2010, 2011). The above discussed powered ankle-foot orthosis is powered by the SEA.

#### Motor Actuator

A motor is often used as a common actuator in ankle rehabilitation robots. For ambulatory individuals, soft robots have contributed to a more natural man-machine interaction, and have minimized the disruption of stroke survivors during the natural dynamics of walking. The most recent innovative design is a soft exosuit (Bae et al., 2015, 2018a,b; Awad et al., 2017b). Awad et al. (2017b) reported that a soft robotic exosuit designed by Harvard University was placed over the paretic limb to enhance forward propulsion and ground clearance, contributing to more normal walking post-stroke. An exosuit transmits mechanical power generated by the actuator to the wearer through the interaction of garment-like, functional textile anchors and cable-based transmissions. The overall mass of the exosuit is only 0.90 kg. The soft exosuit was developed to assist paretic ankle dorsiflexion and plantar flexion.

The Anklebot which was actuated by two linear actuators, has been used for gait rehabilitation post stroke (Roy et al., 2007, 2009, 2011, 2013, 2014; Forrester et al., 2016). The dorsiflexor/plantar flexor torque is generated when both dc-motor pull/push in simultaneous directions, while the actuators move in opposing directions, and creates the inversion/eversion rotational torque. The design is similar to the robotic gait trainer (RGT). However, MIT's Anklebot has some disadvantages such as being heavy, its bulky size, and being tethered. Recently, a lightweight and autonomous ankle robot has been developed for gait training of chronic stroke survivors (Yeung et al., 2017, 2018). The ankle robot is compact, lightweight, and portable.

Additionally, an Electro-hydraulic ankle-foot orthosis (EHO) has been developed by the Laval University (Noel et al., 2009; Blanchette et al., 2014). The custom-design uses a hybrid drive system which includes pneumatic, hydraulic and electric systems. More specifically, the EHO is characterized by its high power and light weight. Moreover, Tanida et al. (2009), Kikuchi et al. (2010) proposed the intelligently controlled ankle-foot orthosis (I-AFO) powered by compact magneto-rheological fluid brakes (MRB). Additionally, the portable powered ankle-foot orthosis, which was designed by the University of Illinois, provides plantar flexor/dorsiflexor torque assistance through a bidirectional pneumatic rotary actuator.

Ankle robots can also be divided into untethered and tethered devices. A few of the presented ankle rehabilitation robots are portable devices (Shorter et al., 2011a; Awad et al., 2017b; Choi et al., 2018; Yeung et al., 2018). Portable devices can be used to assist impaired users in daily life activities. It has been found that SEA and motors are common actuators in portable ankle robots. Moreover, the small size and light weight of the systemic component is a key requirement in the portable robot. In contrast, some tethered devices which are suitable for the gait rehabilitation of stroke survivors in hospitals or rehabilitation centers were designed in studies (Blanchette et al., 2014; Bae et al., 2015; Awad et al., 2017b). The advantage of tethered robots is that they do not add heavy components to the body which results in a lower negative impact for users.

Of those discussed above, the three main types of actuators all have benefits and limitations. PMA has the advantage of a high torque-to-weight ratio and compliance, but in portable systems, the compressed air generator restricts their potential use. SEA is a popular actuator choice in exoskeletons. This is due to advantages including high control precision, low impedance, storing energy, and high back-drivability. However, SEA is limited by the large volume, heavy mass, and complicated structure. Moreover, since the mass, size, and output torque are suitable for most applications, the motor plays the largest role in exoskeletons. Nevertheless, a long power supply provides to the series elastic actuator (SEA), and the motor has traditionally been stated as one issue in the development of portable ankle exoskeletons.

Although the rigid ankle exoskeletons discussed above improve mobility and independence of stroke survivors, it has some drawbacks. Since the rigid ankle exoskeleton adds a burden to the survivor's lower extremity, this will inevitably limit the gait kinematics, resulting in a slow and inadequate gait. Moreover, if the exoskeleton's rigid joints misaligns with the user's biological joints, it can give rise to uncomfortable stress on the soft tissue and bones (Bae et al., 2015). In contrast, soft exoskeletons hold some tremendous advantages such as compliance, natural interaction, adaptation, light weight, small size, less energy used, and is easier to wear. However, it has some demerits. Cable length and routing are not reconfigurable for different survivors with various body types and different paretic sides because the Bowden cable is fixed in series with the actuation system. The soft ankle exoskeleton also has difficulties in transferring power from the area of the body to the ground and motors and sensors are also difficult to mount.

### Gait Event Detection

The powered exoskeleton applies torque during three regions: (1) weight acceptance, dorsiflexor torque is used to control the deceleration of the forefoot; (2) late stance to pre-swing, a propulsive plantar flexor torque is applied to increase propulsion; (3) swing, the ankle exoskeletons provide dorsiflexor torque assistance to allow for the toe clearance (Morris et al., 2011).

As described above, the identification of the specific gait events is key for the user, which can be used to trigger the functional assistance created by the powered exoskeletons. To detect the above, specific gait events are mainly based on measurements from onboard sensors. Based on different sensor information, they could be categorized into three main groups as follows:

1. **Kinetic information**: foot switches or foot pressure insoles;
2. **Kinematics information**: angle sensors;
3. **Muscle motility information**: EMG-based sensors.

#### Kinetic Information

In the works presented in Kim et al. (2007), Tanida et al. (2009), Shorter et al. (2011a), Park et al. (2014), foot switches or foot pressure insoles were utilized to identify and judge gait events. Once sensor magnitudes located in the heel and the metatarsal exceeded the predefined thresholds, gait events are detected. It is noted that force sensor thresholds are adjusted for each user to determine event boundaries during the gait cycle. Moreover, different ankle exoskeletons use a different number of sensors. Most commonly, two force sensors are embedded in the forefoot and heel, respectively, to identify toe off (TO), and heel strike (HS).

#### Kinematics Information

Awad et al. (2017b) reported that kinematic information measured by gyroscope sensors identified gait events to control the assistance time of the exosuit. Combining the detection ankle kinematic and kinetic information of all kinds of sensors can enhance the accuracy of the gait phase detection to make sure that the exoskeleton robot will work effectively and reliably (Blaya and Herr, 2004; Yeung et al., 2017; Choi et al., 2018). It was found that an inertial measurement unit and two force sensors could be used to identify the swing phase and stance phase and three walking modes, namely level walking and stair ascent and descent (Yeung et al., 2017).

#### Muscle Motility Information

Ferris et al. (2006) reports that the soleus electromyography (EMG) signal activates the PMA to generate plantar flexor assistance and the tibialis anterior EMG signal controls the PMA to produce dorsiflexor assistance. More specifically, when the soleus EMG signal magnitudes exceeds the predefined threshold, the control system completely inhibits the activation of the PMA applied to the dorsiflexor motion. Additionally, Joshi et al. (2013) separated eight different phases of gait by utilizing the lower limb EMG signal, which is based on Bayesian Information Criteria (BIC), standard feature extraction methods, and a Linear Discriminant Analysis (LDA) classification algorithm. However, Taborri et al. (2016) suggest that a method based on threshold rules applied on electromyography (EMG) signals performs better than machine-learning algorithms.

In short, mixed sensing techniques which combine kinematic and kinetic information could detect motion intention in a multimodal condition, namely level walking, stair ascent and descent, and ramp ascent and descent. As such, this technique embedded in a portable powered ankle exoskeleton can contribute to the daily life of stroke survivors.

The gait event detection technique may not be robust enough in over-ground walking, as it depends on identifying heel strikes and the foot-flat phase during the gait cycle. Nevertheless, we also observed that some stroke survivors land with the mid-foot rather than walk with a heel strike. Survivors often utilize “vaulting” compensations to reduce foot-drop (Kerrigan et al., 1997), eliminating the foot-flat phase. Therefore, this technique may not be suitable for post-stroke populations. Bae et al. (2018b) reported that the new gait detection algorithm used foot angle and angular velocity measured by foot IMUs to recognize paretic and nonparetic toe-offs, and nonparetic mid-swing. The results showed that the new gait detection algorithm was implemented to improve gait event detection reliability, compared with previous gait event detection algorithms which detected heel strikes and foot flat phases (Bae et al., 2015).

### Control Strategies

The human-robot physical interaction must be appropriately controlled so that the user's safety is ensured. Control strategies for the wearable ankle robot were developed for survivors to recover muscular strength and lost ranges of motion (Jamwal et al., 2015; Khalid et al., 2015; Hussain et al., 2017). According to the different signals that obtain from the initiative intention, the control strategies between robot and survivors normally fall under three categories: position control, force control, and EMG signal control.

#### Position Control

The position control method is trajectory-tracking control, which is to drive the patient's foot to move about on the reference trajectories with the help of the ankle robot. These reference trajectories data were normally measured from the healthy limb or healthy subjects, using the motion sensors in the biomechanics labs (Andersen and Sinkjaer, 1995; Wheeler et al., 2004; Ward et al., 2007, 2010; Shorter et al., 2011a; Park et al., 2014; Awad et al., 2017b).

#### Force Control

A force signal is produced by limb contraction and interactions with a mechanical structure. Compared with the EMG signal, the force signal has better determinacy, which can reflect the motion intention of the patient better, so the control based on force signal is feasible and relatively steady. Force control schemes were found in some studies (Tanida et al., 2009; Blanchette et al., 2014; Choi et al., 2018), however, position control and force control might not be the most appropriate control scheme for robots with a medical purpose, which mainly requires dynamic interaction. To solve this problem, the impedance/admittance control scheme was proposed in the robot control field (Hogan, 1985). Since then, impedance control has gained extensive application (Chiaverini and Sciavicco, 1993; Ziren and Goldenberg, 1995; Seraji and Colbaugh, 2002). Impedance control schemes were found in multiple studies (Blaya and Herr, 2004; Roy et al., 2009, 2011, 2013, 2014; Forrester et al., 2016). The impedance control scheme provides a natural, comfortable, and safe touch interface, effectively avoiding secondary damage. An additional advantage of impedance control is that the achievement of impedance control was independent of prior knowledge (Tsoi and Xie, 2009).

#### EMG Signal Control

An EMG signal is the electrical activity produced by the skeletal muscle (De Luca, 1997; Robertson et al., 2013; Zhang et al., 2017; Li et al., 2018). To effectively extract motor control command from myoelectric signals, substantial work has been carried out (Nurhanim et al., 2014). Several wearable ankle rehabilitation robots have been controlled by EMG signals (Ferris et al., 2005, 2006; Sawicki et al., 2005, 2006; Cain et al., 2007; Takahashi et al., 2015; Koller et al., 2018). EMG signal control has been employed by the University of Michigan's AFO (Ferris et al., 2006; Cain et al., 2007). Ferris et al. (2006) proposed that the soleus electromyography (EMG) signal activated the PMA to generate plantar flexor assistance and the tibialis anterior EMG signal controlled the PMA to produce dorsiflexor assistance. Cain et al. (2007) demonstrated that proportional EMG control led to a larger decrease in muscle activation and gait kinematics closer to normal than footswitch control. Compared with the force signal, EMG has the following advantages: (1) the acquisition of EMG is simple; (2) use of the EMG signal can identify finer movements than force signal; (3) the interactive control based on EMG has more flexibility, which can realize the control of the diseased limb through the healthy limb according to the coordination of the body movement. Nevertheless, the myoelectric signals of stroke survivors may be weakened. Additionally, the ankle muscles post stroke become too weak or paralyzed to generate abnormal muscle activation (Wright et al., 2014). Furthermore, the myoelectric signals are affected by electrode-skin conductivity, improper electrodes alignment, fatigue, and the interaction between nearby muscles (Fleischer and Hommel, 2008; Tucker et al., 2015). The EMG signals are also non-stationary during dynamic activity, which requires the utilization of pattern recognition techniques (Souza et al., 2014). It is usually necessary to calibrate each time the device is installed in practical use (Dawley et al., 2013).

### Performance Evaluation

In this paper, a total of 19 studies assessed the effects of various ankle rehabilitation robots on impaired subjects, which included 97 stroke survivors, two plantar flexor impaired subjects, and four dorsiflexor impaired subjects (Table 3). As shown in Table 3, there is no agreement on outcome measures. Ward et al. (2010) designated a list of performance indicators to evaluate the effect of the ankle robot on stroke survivors. Young and Ferris (2017) mentioned that a 6 min walking test (SMWT) could be used as a clinical measure in stroke survivors. A higher walking speed indicates better clinical outcomes because walking speed is closely related to social mobility. The most common criteria to assess the clinical performance of ankle rehabilitation robots on stroke survivors is summarized in Table 4. Paretic peak dorsiflexion angle during the swing, propulsion on the paretic side during push-off, and a 6 min walking test are the selected outcome measures used to evaluate the clinical effects of the robot in reducing foot slap, foot-drop and improving propulsion, and muscle activation.

**Table 3:** Review studies of performance evaluation of wearable ankle rehabilitation robot.

**References**	**Design**	**Subject**	**Characteristics**	**Intervention**	**Comparisons**	**Outcome measure**	**Outcome**
Blaya and Herr, 2004	Case control study	5	2 Dorsiflexor impaired (62 years, 87.25 kg, 178 cm)	NO assistAFO assistPowered assist	NO assist AFO assist Powered assist Healthy	Kinematic and kinetic gait	↑ Df in swing; ↑ Pf in stance; ↓Occurrence of foot slap; ↓Step length and step time asymmetry
			3 Healthy survivors (66.6 years, 78.6 kg, 171.7 cm)	/			
Kim et al., 2007	Case study	1	Hemiplegic patient (52 years, 68.5 kg, 166.5 cm)	NAFO,HAFO,AAFO (training:4 weeks, test: 30 min)	Pre-post	ROM of AAFO, Temporal-spatial parameters, Angles of the ankle and the knee	↑ Walking speed and cadence; ↑ Dorsiflexion RoM; ↑ Plantar flexion in push-off
Kim et al., 2011	Case study	3	Hemiplegic survivors (51 ± 2.3 years, 63.5 ± 5.7 kg, 163.5 ± 4.2 cm)	NAFO,HAFO,AAFO (training:4 weeks, test:30 min)	Pre-post	Temporal-spatial parameters; Joint angles	↑ Walking speed and cadence; ↑ Dorsiflexion RoM; ↑ Plantar flexion in push-off
Ward et al., 2007	Case study	1	Stroke survivor (22 years)	Training(16 sessions,8 weeks,60 min/2/week)	Pre-mid-post	SMWT; Timed get up and go	↑ SWMT; ↑ three-meter forward and backward tests; ↑“get-up-and-go” test.
Ward et al., 2010	Case study	3	Stroke survivors (52 years, 80.1 kg, 171 cm)	Over-ground walkingTreadmill walking(NPAFO/PAFO)	Pre-post	Kinematic and kinetic gait parameters; Ankle angle; SMWT	↑SWMT; improved kinematics;
Ward et al., 2011	Case Study			Training (9 sessions,3 weeks, 34 min/3/week)	Pre-post	Kinematic and kinetic gait parameters; Ankle angle;	↑ Cadence; ↑ Ankle range of motion;
Shorter et al., 2011a	Case control study	4	3 Healthy subjects (26 ± 4 years, 79 ± 6 kg, 187 ± 7 cm) 1 plantar flexion impaired subject(51 years, 86 kg, 175 cm)	For disabled(1 min NAFO-1 min NPAFO-1 min PPAFO x3 conditions)For non-disabled(1.5 min NAFO-1.5 min NPAFO-1.5 min PPAFOx3 conditions)	NAFO NPAFO PPAFO (x3 conditions)	Kinetic gait parameters; Muscle activation; Ankle angle	For nondisabled, ↓ Tibialis anterior activation; For disabled, ↑ plantarflexion;
Shorter et al., 2011b	Case study	2	1 Plantar flexor impaired subject (51 years, 86 kg, 175 cm) 1 Dorsiflexor impaired subject (37 years, 62 kg, 157 cm)	No AFOUnpowered PPAFOPowered PPAFO	NAFO PPAFO UPPAFO	Kinematic and kinetic gait parameters; Ankle angle	↓Df; improved Push-off phase
							↓ Occurrence foot-drop; better foot positioning heel strike;
Roy et al., 2013	Case study	1	Dorsiflexor impaired subject	NO assist; Anklebot–assist (18 sessions, 6 weeks, 3/week, 40 min/session)	Pre-post-follow up	Ankle angle	↑ Df in swing
Forrester et al., 2016	RCT	26	Stroke survivors, Treadmill robotic (*n* = 14, 59.5 ± 3.6 years, 81.5 ± 4.2 kg, 168 ± 3 cm); Seated robotic (*n* = 12, 56.8 ± 3.2 years, 85.0 ± 3.7 kg, 170 ± 3 cm)	18 Sessions (3x weekly; 6 weeks), one session:Treadmill robotic training(60 min)Seated robotic training (60 min)	Pre-post-follow up	Kinematic and kinetic gait	In TMR group, ↑paretic single support duration;↑peak swing angle;↑propulsive impulse on paretic side;
Blanchette et al., 2014	Case study	6	Stroke survivors (66.7 years, 77.7 kg, 169.3 cm)	NO EHO EHO	Pre-mid-post	Spatiotemporal gait parameters; ankle and knee kinematics; activity of TA and Soleus	↑TA in 4 of 6 subjects; ↑ Df during the swing in 3 of 4
Takahashi et al., 2015	Case study	5	Stroke survivors (61.2 ± 14.3 years, 98.6 ± 17.4 kg, 179 ± 9cm)	NoEXO (5 min) UnPOW (5 min) POW (5 min)	NoEXO UnPOW POW	Peak paretic ankle plantarflexion moment; symmetry of GRF propulsion impulse; net metabolic power	↑Paretic plantarflexion moment; ↓net metabolic power; ↓paretic soleus activation
Awad et al., 2017a	Case study	8	Stroke survivors (47.8 years)	Unpowered exosuit (8 min) Powered exosuit (8 min)	Unpowered-powered	Spatiotemporal parameters; Swing phase kinematic parameters;	↓ Hip hiking and circumduction; ↑Ankle dorsiflexion angle in swing
Awad et al., 2017b	Case study	9	Stroke survivors (49 ± 4 years, 77.8 kg, 173.1 cm)	Unpowered exosuit (8 min) Powered exosuit (8 min)	Unpowered- Powered 2 different onset timings	Peak paretic propulsion; Interlimb propulsion symmetry; Swing phase ankle Df angle; Metabolic burden;	↑Ankle dorsiflexion angle in swing; ↓Asymmetry; ↓ metabolic burden
Bae et al., 2015	Case study	3	Stroke survivors (50.67 years, 81.2 kg, 176cm)	Baseline Powered	Baseline-Powered	Spatiotemporal parameters;	↑Step and stance time symmetry; ↑Propulsion symmetry; ↓ circumduction
Bae et al., 2018a	Case study	7	Stroke survivors (49 ± 4 years, 72.96 kg, 172.3 cm)	Unpowered exosuit (8 min) Powered exosuit (8 min)	Unpowered- Powered	COM power; Joint power; Metabolic power;	↑ Symmetry ankle power generation; ↑ Symmetry body CoM power generation; ↓Metabolic cost
Bae et al., 2018b	Case study	3	Stroke survivors (64 years, 84.3 kg, 176.3 cm)	NOEXO(5 min) EXO_ON1 (optimized control, 5 min) EXO_ON2 (previous control,5 min)	NOEXO; EXO_ON1 EXO_ON2 Healthy	Kinematic and kinetic gait;	↑ Propulsion symmetry; ↑ Ground clearance; ↓ Metabolic cost;
Yeung et al., 2018	RCT	19	Chronic stroke survivors, robotic group (*n* = 9, 54.2 ± 13.0 years), Control Group (*n* = 10, 61.2 ± 10.6 years)	10 min level walking 10 min stair ascend 10 min stair descend 2/week/20-session	Sham Group RoboticGroup Pre-post-Follow-up	Main outcome: FACSecondary outcome: FMA, MAS, BBS,10 MWT,SMWT, Spatial-temporal, kinetic, and kinematic gait parameters	In Robotic Group, ↑ Gait independency and walking speed; ↑ Confidence in paretic limb loading response; In Sham Group, ↓affected leg range of motion during the swing.
Yeung et al., 2017	Case study	3	Chronic stroke survivors 58–72 years	NoRobot, NoPower, Powered; 10 min level walking 10 min stair ascend 10 min stair descend	NoRobot- NoPower- Powered	Ankle angle	↓Occurrence foot-drop; No enhancing the gait propulsion.

*AFO, Ankle-foot orthoses; NAFO, gait without AFO; HAFO, hinged AFO; AAFO, active AFO; PAFO, powered AFO; NPAFO, unpowered PAFO; PPAFO, portable powered AFO; FAC, functional ambulatory category; FMA, fugl-meyer assessment; MAS, modified ashworth scale; BBS, berg balance scale; 10 MWT, timed 10-meter walk test; SMWT, 6-min walk test; ROM, ankle range of motion; EHO, electro-hydraulic ankle-foot orthosis; EXO, exoskeleton; COM, center of mass; GRF, ground reaction force; TA, tibialis anterior; Df, Dorsiflexion; Pf, Plantar flexion; Is, inversion, Es, eversion*.

**Table 4:** Overview of outcome measure.

**Outcome measure**	**Metrics**
Temporal-spatial parameters	Step cadence; step length; step time; gait symmetry; walking speed
Kinematic gait parameters	Ankle range of motion (ROM); Maximum plantar flexion angle; Swing phase ankle dorsiflexor angle
Kinetic gait parameters	Anterior-posterior ground reaction force (GRF); Vertical ground reaction force (GRF); Maximum ankle torque; Joint power
Assessment scale	Functional Ambulatory Category (FAC) Timed 10-Meter Walk Test (10 MWT); 6-min Walk Test (SMWT); Timed get up and go. Modified Ashworth Scale (MAS); Berg Balance Scale (BBS)
Muscle activation	Tibialis anterior (TA); Soleus; gastrocnemius
Metabolic power	Metabolic cost of walking

#### Muscle Activation

Studies (Shorter et al., 2011a; Takahashi et al., 2015) evaluated the effect of ankle rehabilitation robots on the muscle activation of the lower limb of survivors. In Takahashi et al. (2015), a powered ankle exoskeleton was used for five stroke subjects to walk three sessions for 5 min each session. The results showed that the soleus activation of the paretic side of the three subjects during the propulsion phase was decreased with the powered assistance compared to unassisted walking. Meanwhile, in the powered assisted walking test, the exoskeleton increased the paretic plantar flexion torque by 16% relative to the unassisted walking condition. However, in Shorter et al. (2011a), it can be seen that the tibialis anterior activation in stance and swing phase was reduced during assisted walking trials for non-disabled subjects. Moreover, Kao and Ferris (2009) studied the effect of an active dorsiflexion assist orthosis that was proportionally controlled by tibialis anterior electromyography on the muscle activation of the neurologically intact subjects. It was shown that in the continuous group, the amplitude of tibialis anterior EMG during the swing-to-stance phase transition was reduced by 28%, in contrast, the amplitude of tibialis anterior EMG during the stance-to-swing phase in both groups was not decreased. Therefore, the results only partially supported their hypothesis that the subject's tibialis anterior muscle activity is reduced during walking with a powered dorsiflexion assist orthosis which is proportionally controlled by the tibialis anterior EMG.

Furthermore, Young and Ferris (2017) reported that exoskeletons were to reduce muscle recruitment of the lower limb during walking. The EHO (Blanchette et al., 2014) has been developed to assess the residual adaptive capacity of ankle dorsiflexor when the paretic limb of stroke survivors was added to a perturbation. An interesting result was reported that the mean amplitude of tibialis anterior (TA) in four of the six participants was significantly increased after the walking period with the perturbation. Moreover, modifications in tibialis anterior (TA) activation in three of the four participants persisted after perturbation removal.

From the results mentioned above, it can be found that for healthy subjects, reducing plantar flexion and dorsiflexion muscle recruitment walking with ankle exoskeletons reduces the user's metabolic cost of walking. In contrast, for stroke survivors, there is no consensus that the ankle exoskeletons can reduce dorsiflexion and plantar flexion muscle activity.

#### Peak Dorsiflexion Angle on Paretic Side During the Swing

Post-stroke paresis of the ankle musculature causes a weak ability of the active dorsiflexion during the swing phase. The ankle rehabilitation robot can enhance the ability of ankle dorsiflexion of the paretic limb and ground clearance, which reduces the risk of falling. Dorsiflexion angular range during the swing needs to be measured to quantify the reduction of the second major complication of foot-drop. The comparison of peak dorsiflexion angle during the swing is listed in Table 5.

**Table 5:** Peak dorsiflexion angle during the swing.

**References**	**Comparisons condition**	**Peak angle in swing**
Forrester et al., 2016 (TMR)	Post-pre	**+5.3°**
	Follow-up-pre	**+5.1°**
Roy et al., 2013	Post-pre	**+5.1°**
	Follow-up-pre	**+5.5°**
Blanchette et al., 2014	Post-pre	3 subjects(**+6.6°**, +4.2°, and +1.6°)
Awad et al., 2017b (TMR)	Unpowered-powered	**+5.33°**
Awad et al., 2017b (OGT)	Unpowered-powered	+4.9°
Awad et al., 2017a	Unpowered-powered	+4.78°
Blaya and Herr, 2004	Unpowered-powered	+37–200%

*TMR, treadmill robotic training; OGT, over-ground training*.

*An increase of 5 degree is marked in boldface*.

Studies (Roy et al., 2013; Forrester et al., 2016; Awad et al., 2017b) showed the improvement of the dorsiflexion angle on the paretic side during the swing after rehabilitation training. Forrester et al. (2016) reported that the peak dorsiflexion angle on the paretic side of stroke survivors during the swing in the treadmill robot training group was increased, with gains sustained at a follow-up test. A similar effect was obtained in Roy et al. (2013), achieving bigger gains in a follow-up test. Awad et al. (2017b) assessed the peak dorsiflexion angle on the paretic side during the swing in a treadmill group for unpowered vs. powered over-ground training. Compared with the over-ground group, the treadmill group achieved a greater improvement of the peak dorsiflexion angle on the paretic side. An increase of 5° (bold from Table 5) in the dorsiflexion movement was usually regarded as clinically significant (Rose et al., 2010). However, an increase of the dorsiflexion angle reported in Blanchette et al. (2014), Awad et al. (2017a), Awad et al. (2017b) was <5°. The results may be related to a single session of walking with powered ankle exoskeletons. In contrast, multiple sessions of training contributed to statistically significant increases in the dorsiflexion angle on the paretic side (Roy et al., 2013; Forrester et al., 2016). Therefore, gait rehabilitation post-stroke may benefit from multiple sessions of gait training using an ankle rehabilitation robot. By comparing immediate effects between unpowered and powered ankle rehabilitation robot, it would be more valuable to assess statistical significance before, and after the rehabilitation training of multiple sessions with an ankle rehabilitation robot. In particular, follow-up evaluation is warranted to validate the sustainability of gait recovery.

Furthermore, the active range of motion in the dorsiflexion of a seated ankle rehabilitation robot was also reported in Forrester et al. (2016), showing that the treadmill robotics (TMR) group made significant progress and continued to improve during the 6 w period after rehabilitation training. We believe that an active range of motion in dorsiflexion of standing posture without any assisted device might be a key and valuable evaluation indicator.

#### Peak Propulsion on Paretic Side During Push-off

Ankle rehabilitation robots designed for assisting propulsion on the paretic side could reduce propulsion asymmetry and facilitate more normal walking in patients after stroke. Moreover, the improvement of propulsion on the paretic side plays a decisive role in the improvement of walking speed.

The results reported in Table 6 show the effect of powered exoskeletons on the ankle paretic propulsion, propulsion asymmetry and propulsion impulse of stroke survivors. All the reported studies found an improvement in propulsion on the paretic side, propulsion asymmetry and propulsion impulse in Table 6. However, peak paretic propulsion during assisted push-off with powered exoskeleton was reduced in the sham group vs. the robotic group after 20 training sessions in Yeung et al. (2018). One of the possible reasons that might explain this result is traditional rigid ankle-foot orthoses (AFOs) in the sham group, which limits normal push-off and reduces gait adaptability (Vistamehr et al., 2014). Furthermore, it can be seen that compared to unassisted walking, the exoskeleton did not affect the percentage of paretic propulsions in stroke survivors walking with the powered assistance (Takahashi et al., 2015). One possible reasons is the low sample size. Other factors may include suboptimal timing of exoskeleton driving and insufficient adaptation when using the exoskeleton. Only a few studies that have been performed with powered exoskeletons assessed the effect on propulsion asymmetry (Bae et al., 2015; Awad et al., 2017b) in Table 6. It has been shown that walking assisted push-off with powered ankle exoskeletons can reduce propulsion asymmetry.

**Table 6:** Propulsion on paretic side during push-off.

**Reference**	**Propulsive force**	**Propulsion asymmetry**	**Propulsion impulse**
Yeung et al., 2018 (Sham group)	**−0.02** N/kg	/	/
Yeung et al., 2018 (Robotic group0	+0.10 N/kg	/	/
Awad et al., 2017b (tethered)	+11%	−20%	/
Awad et al., 2017b (Untethered)	+13%	−16.3%	+14%
Forrester et al., 2016 (TMR)	/	/	+12.1 N.s(post-pre), +19.2 N.s(follow-up-pre)
Forrester et al., 2016 (SRT)	/	/	–**1.4** N.s(post-pre), +2 N.s (follow-up-pre)
Shorter et al., 2011b	+25 N	/	/
Bae et al., 2015		−7.15%	/

*TMR, treadmill robotic training; OGT, over-ground training; SRT, Seated robotic training*.

*An reduction of propulsice force and impulse is marked in boldface*.

Forrester et al. (2016) verified that the ankle robot combined with a treadmill was more significantly effective in increasing paretic push-off impulse than the seated training after 6 w of rehabilitation training. Therefore, for chronic stroke survivors, a treadmill combined with ankle robot training improves gait function more effectively than robots that focus on ankle training.

#### Walking Capacity

Stroke survivors are characterized by hemiplegia gait, causing a slow, metabolically inefficient gait and an increased risk of falling. In Young and Ferris (2017), it is suggested that in these post-stroke populations, the clinical measurement method is the walking speed, usually using the 6 min walk test (SMWT). Higher priority walking speeds indicate better clinical outcomes because walking speed is closely related to social mobility. Yeung et al. (2018) reported that after the 20-session gait training, the SMWT of robotic and sham groups showed a certain extent of improvements in walking capacity and endurance, which improved both + 5.7 m (post-pre), and + 22.2 m (follow-up-pre) in sham groups, and increased + 16.9 m (post-pre) as well as + 41.5 m (follow-up-pre) in robotic groups. The 17% increased walking capacity was greater than the minimal clinically important difference (MCID) of SMWT for walking endurance, which is a 11.5% proportional change in walking distance for stroke survivors (Tang et al., 2012).

Studies (Ward et al., 2007, 2010) assessed the effect of an ankle rehabilitation robot on walking capacity of a stroke survivor. In Ward et al. (2007), it is reported that a stroke survivor obtained higher gains from RGT (Robotic Gait Trainer) across the 16-sessions of gait training, contributing to an increase in SMWT. Moreover, the results from SMWT for subject 1 and 3 between the pre-and post-intervention test showed improvements (Ward et al., 2010). Both ankle rehabilitation robots have been assessed on stroke survivors for at least three consecutive weeks in gait training sessions. Although the two clinical studies did not have control trials and the sample size was small, the researchers found that the longer the walking distance of the SMWT, the better the clinical outcomes.

## Discussion

### Challenges of Wearable Ankle Robots

During the last few decades, ankle rehabilitation robots have been shown to have great potential in assisting or rehabilitating the ankle joints of stroke survivors to eventually improve gait function. However, some challenges limit its extensive use.

Onboard actuation has been a crucial issue throughout ankle rehabilitation robot development. Actuators play the key role in ankle rehabilitation robots and determines the assistance torque provided by the robot in gait training. Sufficient torque can fully provide functional assistance in gait training, thus promoting gait rehabilitation of stroke survivors. Moreover, the weight of the powered ankle exoskeleton is a critical factor. Veale and Xie (2016) proposed that to be comfortably worn, the maximum weight added on the user's segments should not exceed 15 and 1.25% of the user's body weight when placed on the torso and each foot, respectively. Furthermore, Rossi et al. (2013) demonstrated that adding a weight of 2.5 kg on the leg in a short period did not change the kinematics of the lower extremities. It has been shown that 3.6 kg (MIT's Anklebot) unilateral loading did not significantly alter the gait pattern of chronic stroke survivors (Khanna et al., 2010). Nevertheless, Yeung et al. (2018) reported that longtime wear of a weight of 0.5 kg at the affected ankle would still change the gait pattern even after the stroke survivor removed the robot. The heavy weight of the ankle rehabilitation robot would increase the burden on the lower limbs of stroke survivors, change the gait pattern, and adversely affect gait rehabilitation. Therefore, lightweight and high output torque actuators need to be further developed.

More research should be performed to develop the mechanism design of inversion/eversion. Mattacola and Dwyer (2002) clinically demonstrated that both plantar flexion/dorsiflexion and inversion/eversion were the main motions performed during walking. Moreover, inversion is also a typical characteristic of post stroke gait and the main cause is inversion muscle spasticity and eversion muscle weakness. However, most of the existing ankle rehabilitation robots provide only dorsiflexion and plantar flexion assistance. Hence, we believe that ankle rehabilitation robots need to provide not only dorsiflexion and plantar flexion assistance, but also inversion and eversion assistance, so as to enhance the muscle strength of inversion and eversion and to promote a more comprehensive gait rehabilitation of stroke survivors.

The other major challenge is gait event detection of the wearable ankle rehabilitation robot. Gait event detection is key for gait rehabilitation of stroke survivors, which can be used to trigger the functional assistance. At present, the gait event detection technique may not be suitable for post-stroke populations. Therefore, on the one hand, more studies are be needed to improve the understanding of the pathology and walking patterns of stroke survivors. On the other hand, it is crucial for researchers to propose new gait event detection techniques adapted to stroke survivors. Furthermore, mixed sensing technology can be applied to identify and judge the gait event for stroke survivors. Only by accurately identifying the complicated abnormal gait phase of stroke patients can the functional assistance time of the limbs on the side of paresis be guaranteed, so as to fully guarantee the efficient gait rehabilitation.

A significant issue that remains is which type of controller is more suitable for assisting stroke survivors to maximize the benefits of ankle rehabilitation robots. The control strategies are an integral part of the wearable ankle robots and aims to create a safe, comfortable and natural human-computer interaction environment. Controllers for different exoskeletons vary widely, and few studies directly test different controllers on the same hardware. This makes it very difficult to assess or compare the effectiveness of one controller to another. Some studies demonstrated that a proportional myoelectric control brings about a larger decrease in muscle activation and gait kinematics closer to normal than footswitch control (Cain et al., 2007; Koller et al., 2015, 2017). However, while these results have been assessed with healthy subjects, there is a lack of research performed on stroke survivors. Future studies should be conducted to compare the effects of different types of controllers on stroke survivors. Additionally, Marchal-Crespo and Reinkensmeyer (2009) proposed that providing too much assistance has negative consequences in terms of control strategies. Therefore, according to the real-time performance of the ankle joint, it is only necessary to help the patient if needed, or to systematically reduce its assistance during the recovery process. The application of artificial intelligence was also considered and embedded in some designs, to customize the survivor's needs in different recovery stages (Tsoi and Xie, 2009). The most suitable control strategy can ensure a safe and comfortable human-computer interaction in gait training, stimulate patients' enthusiasm for rehabilitation, and thus promote the effect of gait rehabilitation.

The majority of the included studies had small sample sizes, which may have limited the significant effect. Furthermore, few randomized controlled trials (RCTs) have been conducted to assess the clinical performance of powered ankle exoskeletons for stroke survivors. Yeung et al. (2018) reported that robot-assisted gait training could increase independent walking capacity and help stroke survivors promote confidence in weight acceptance. Forrester et al. (2016) demonstrated that as for chronic stroke survivors, a treadmill combined with ankle robot training improves gait function more effectively than robots that focus on the ankle joint. Awad et al. (2017b) assessed the immediate effects of a robotic exosuit actively assisting the impaired limb of chronic stroke survivors during over-ground and treadmill walking. Future studies should be performed to assess the effect of ankle rehabilitation robots on stroke survivors during weight-supported treadmill training and over-ground training, respectively. Additionally, more research should be conducted with follow-up tests to assess whether the improvement obtained by powered ankle exoskeleton was maintained or not. In other words, a multicenter randomized controlled clinical trial should be conducted to propel the clinical application of powered ankle exoskeletons in the future.

## Limitations

In this article, we hypothesized that all researches used different subjects, but since some studies were performed at the same place, we were not sure whether unrelated study populations were employed. However, there may be other studies where the robot or ankle was not identified as a key term in the review. This study only included articles from 1995 onwards, because the occurrence of a wearable ankle rehabilitation robot was very limited before then. Moreover, we included published journal and conference papers with a clear description of the wearable ankle robot, but did not include those written in languages other than English. Therefore, some studies may not have been included on this basis, resulting in a potentially incomplete search.

## Conclusion

In this paper, the classification of ankle rehabilitation robots, actuators, gait event detection, control strategies, and performance evaluation are reviewed. In terms of the actuator, motors are popular in portable powered ankle exoskeletons. Furthermore, lightweight, small size, and high output torque actuators need to be developed. As for gait event detection techniques, mixed sensing technology which combines kinematic with kinetic information is effective to detect the gait event of stroke survivors. In this article, all the selected clinical studies showed improvement in the dorsiflexion angle in the swing phase and propulsion on the paretic side during push-off after a period of robot-assisted ankle rehabilitation training. Therefore, it can be seen that the ankle rehabilitation robot can reduce foot slap during weight acceptance and foot-drop during swing, and improve propulsion during push-off, resulting in higher walking speeds. As for the gait rehabilitation of stroke survivors, it is difficult to determine the most effective ankle rehabilitation robot. On the one hand, this is due to different devices or control strategies. On the other hand, this is caused by the fewer randomized controlled clinical trials. Additionally, a lack of universal evaluation criteria is also a reason. In other words, in the near future, a multicenter randomized controlled clinical trial is necessary to enhance the clinical effectiveness of wearable ankle robots.

## Data Availability

All datasets generated/analyzed for this study are included in the manuscript and/or the supplementary files.

## Author Contributions

BS performed the study, analyzed the data, and was responsible for drafting the manuscript. XC helped perform the study and revised the manuscript. ZY and SY participated in the study selection and helped in the data extraction. QW, XZ, and WW analyzed and discussed the results. JW conceived and designed the review. All authors read and approved the final manuscript.

### Conflict of Interest Statement

The authors declare that the research was conducted in the absence of any commercial or financial relationships that could be construed as a potential conflict of interest.
